# Abuse-deterrent formulations and opioid-related harms in North Carolina, 2010-2018

**DOI:** 10.1093/aje/kwae252

**Published:** 2024-08-09

**Authors:** Bethany L DiPrete, Nabarun Dasgupta, G Yeon Oh, Daniela C Moga, Svetla Slavova, Emily Slade, Chris Delcher, Brian W Pence, Shabbar I Ranapurwala

**Affiliations:** Department of Epidemiology, University of North Carolina at Chapel Hill, Chapel Hill, NC, United States; Injury Prevention Research Center, University of North Carolina at Chapel Hill, Chapel Hill, NC, United States; Department of Pharmacy Practice and Science, College of Pharmacy, University of Kentucky, Lexington, KY, United States; Institute for Pharmaceutical Outcomes & Policy, College of Pharmacy, University of Kentucky, Lexington, KY, United States; Sanders-Brown Center on Aging, University of Kentucky, Lexington, KY, United States; Department of Epidemiology and Environmental Health, University of Kentucky, Lexington, KY, United States; Department of Pharmacy Practice and Science, College of Pharmacy, University of Kentucky, Lexington, KY, United States; Institute for Pharmaceutical Outcomes & Policy, College of Pharmacy, University of Kentucky, Lexington, KY, United States; Sanders-Brown Center on Aging, University of Kentucky, Lexington, KY, United States; Department of Epidemiology and Environmental Health, University of Kentucky, Lexington, KY, United States; Department of Biostatistics, University of Kentucky College of Public Health, Lexington, KY, United States; Department of Biostatistics, University of Kentucky College of Public Health, Lexington, KY, United States; Department of Pharmacy Practice and Science, College of Pharmacy, University of Kentucky, Lexington, KY, United States; Institute for Pharmaceutical Outcomes & Policy, College of Pharmacy, University of Kentucky, Lexington, KY, United States; Department of Epidemiology, University of North Carolina at Chapel Hill, Chapel Hill, NC, United States; Department of Epidemiology, University of North Carolina at Chapel Hill, Chapel Hill, NC, United States; Injury Prevention Research Center, University of North Carolina at Chapel Hill, Chapel Hill, NC, United States

**Keywords:** opioid analgesics, postmarketing evaluation studies, study design, opioid prescribing, opioid-related disorder

## Abstract

Abuse-deterrent formulations of opioid analgesics (ADFs) were introduced to reduce opioid-related harms among pain patients, but postmarketing study results have been mixed. However, these studies may be subject to bias from selection criteria, comparator choice, and potential confounding by “indication,” highlighting the need for thorough study design considerations. In a sample of privately insured patients prescribed ADF or non-ADF extended-release/long-acting (ER/LA) opioids in North Carolina, we implemented a version of the prevalent new-user design to evaluate the relationship between ADFs and opioid use disorder (OUD, *n* = 235) and opioid overdose (*n* = 18) through 6 months of follow-up using inverse probability-weighted cumulative incidence functions and Fine-Gray models. The weighted hazard ratio (HR_w_) of opioid overdose among patients initiating ADFs was 0.87 (95% CI, 0.23-3.24) times as high as among patients who initiated, restarted, or continued non-ADF ER/LA opioids. We observed a short-term benefit of ADFs for incident OUD (HR_w_ = 0.58; 95% CI, 0.35-0.93) compared to non-ADF ER/LA opioids in the first 6 weeks of follow-up, but this benefit disappeared later in follow-up (HR_w_ = 1.30; 95% CI, 0.86-1.95). In summary, our findings add to the expanding body of evidence that there is no clear long-term reduction in harm from ADF opioids among patients in outpatient use.

**This article is part of a Special Collection on Pharmacoepidemiology**.

## Introduction

Prescription opioid analgesics were commonly implicated in overdose deaths in the first 2 decades of the 2000s in the United States (abbreviations listed in Table S1),^1^ reinvigorating pharmaceutical engineering innovations intended to reduce crushing (and injection or snorting) of tablets.^2^^-^^4^ Extending back at least to the 1980s, real-world evaluations of so-called abuse-deterrent formulations (ADFs) have consistently shown mixed and limited success in reducing drug-related harms,^5^^-^^13^ in part because patients prescribed the medications are somewhat distinct from those who obtain them from outside the medical system. However, starting around 2010, multiple opioid analgesic formulations were brought to market with tamper-deterring technology, including physical properties that made physical manipulation difficult or led to the release of an opioid antagonist if crushed.^14^ In 2015 and 2017, the US Food and Drug Administration (FDA) provided a Guidance to Industry on the evaluation of these products, including a pathway for label claims.^15^^,^^16^

While premarket evaluation of new putative ADFs is well defined by these guidances, real-world evaluation has proven challenging. Surveillance and localized studies of illicit drug-using populations^17^^-^^33^ have generally found meaningfully lower nonmedical use of prescription opioids with ADF properties, most of which focused on the reformulation of extended-release oxycodone (OxyContin; Purdue Pharma, LP) in the United States. A notable exception was the multimodal suite of studies conducted in Australia.^34^^-^^37^ However, fewer studies have examined the impact of ADF opioids on intended patient populations with painful conditions.^5^^,^^38^^-^^40^

While measuring safety of ADFs in controlled trials with clear endpoints is relatively straightforward, estimating the impact of ADFs in postmarketing epidemiologic studies is more complex.^41^ As highlighted by Turk et al.,^41^ crucial design considerations must be accounted for in pharmacoepidemiologic studies, particularly those that utilize insurance claims data. These studies may be subject to multiple sources of bias due to selection criteria, choice of comparators, and potential confounding by “indication.”^41^^-^^43^ In 2017, the FDA convened a workshop to discuss these methodological issues.^44^ Most studies to date, often industry-sponsored, have focused on 1 ADF or had limited methods to address methodological difficulties.

In this study, we examined the relationship between ADFs and opioid-related harm (eg, opioid use disorder, opioid overdose) using rigorous epidemiologic methods to address issues of confounding by “indication” and choice of comparators that often arise in opioid research. We implemented a study design including patients with variable treatment histories,^45^^,^^46^ comparing patients initiating ADFs to patients initiating, restarting, or continuing non-ADF extended-release/long-acting (ER/LA) opioids to understand the relationship between any ADF use and the risk of opioid-related harm.

## Methods

### Data sources

Data for this analysis were from a large private health insurance provider in North Carolina (NC), United States. The data source included pharmaceutical, inpatient, and outpatient claims and membership files for individuals with (1) a pain diagnosis using the *International Classification of Diseases, Ninth Revision, Clinical Modification* (*ICD-9-CM*) and *International Classification of Diseases, Tenth Revision, Clinical Modification* (*ICD-10-CM*); (2) surgical procedure, defined using *Current Procedural Terminology* (*CPT*) codes; or (3) outpatient opioid analgesic prescription claim, identified using National Drug Codes, from January 1, 2006, through September 30, 2018. Claims data were linked to death records from the NC Department of Health and Human Services using a hierarchical matching algorithm (Figure S1).

This analysis was part of a larger study approved by the Institutional Review Board, Office of Human Research Ethics, University of North Carolina at Chapel Hill.

### Cohort selection and study design

Adult (18-64 years) patients were eligible for inclusion if they initiated ER/LA opioid analgesics (ADF or non-ADF). Patients with a documented history of opioid overdose or opioid use disorder (OUD) based on *ICD-9-CM* or *ICD-10-CM* diagnostic codes in insurance claims (Table S2) were excluded using all-available lookback. Lookback periods could extend before the introduction of ADF ER/LAs (hereafter referred to as “ADF”) in August 2010; however, to improve comparability between groups, we limited the analytic observation period to August 1, 2010, through September 30, 2018.

We implemented a study design similar to the prevalent new-user design^45^^,^^46^ first proposed by Suissa et al^45^ for examining a new drug when a standard treatment already exists, where many individuals initiating this new treatment likely have a history of exposure to the comparator treatment, as is the case with individuals initiating ADFs. This design accounts for the fact that individuals might follow different treatment trajectories when initiating the drug of interest: (1) directly switch from the comparator (ie, non-ADF ER/LA) to the drug of interest (ie, ADF), (2) switch from the comparator to the drug of interest after a treatment gap (ie, delayed switch),^46^ or (3) initiate the drug of interest without prior exposure to the comparator (ie, new initiator).

This design allows us to examine our exposure of interest (ADF use) in relation to our chosen comparator (non-ADF ER/LA) through the lens of a hypothetical trial examining the following research question: “What if patients who initiated ADFs had instead initiated, continued, or re-initiated non-ADF ER/LAs at the time of ADF initiation?”^46^ To examine this question, we created 2 subcohorts: ADF and non-ADF ER/LA (comparator). For the ADF subcohort, we identified individuals’ first ADF outpatient pharmaceutical claim after August 1, 2010, that was not co-filled with a non-ADF ER/LA prescription on the same day (Figure S2). For the non-ADF ER/LA subcohort, we identified the first non-ADF ER/LA claim after August 1, 2010, for individuals who did not have a prior ADF after August 1, 2010. The ADF and non-ADF ER/LA subcohorts were not mutually exclusive; a patient could be exposed to non-ADF ER/LAs before ADF initiation but not vice versa. Individuals with claims for both an ADF and non-ADF ER/LA on the index date were excluded.

The ADF and comparator subcohorts included both traditional new users^47^^,^^48^ of opioid analgesics and individuals with a history of prescription opioid use. To be considered a traditional new user, patients had to have ≥ 6 months of continuous enrollment that included a ≥ 6-month washout period with no evidence of immediate-release (IR) or ER/LA opioid claims before ADF or non-ADF ER/LA initiation (Figure S2). If a patient had evidence of opioid use before ADF or non-ADF ER/LA initiation, they had to meet the 6-month continuous enrollment and washout period requirement before initiating IR or non-ADF ER/LA opioids (ADF subcohort) or IR opioids (non-ADF ER/LA subcohort). We limited analyses to the first ADF or non-ADF ER/LA initiation after July 31, 2010, that met these criteria.

### Outcomes

Outcomes of interest were (1) first of: fatal opioid overdose (identified using *ICD-10* codes for underlying and contributing cause of death in linked death records, Table S3), or nonfatal opioid overdose; (2) diagnosed OUD; and (3) a combined outcome of first of: opioid overdose (fatal or nonfatal) or diagnosed OUD. We treated death from causes other than opioid overdose as a competing risk.^49^^,^^50^

### Follow-up and comparator selection

We defined the index date (baseline) as the date of ADF initiation for patients initiating ADF opioids and the date of the prescription selected as a non-ADF ER/LA comparator (Figures S3-S4).

In defining risk sets and selecting comparators, we included all new ADF prescriptions (ie, all ADF patients) and, for everyone in the ADF cohort, randomly selected 1 non-ADF ER/LA history-based comparator. To account for opioid exposure history and minimize potential selection bias from exclusion criteria in non-ADF ER/LA users with prior opioid exposure (by erroneously excluding incident opioid overdose or OUD outcomes as history events, described in detail by Suissa et al^45^), we arranged ADF index dates by calendar time and then selected 1 non-ADF ER/LA prescription (comparator) with equivalent (1) months since first non-ADF ER/LA (categorized as 0, 1-3, 4-6, 7-9, 10-12, 13-17, 18+ months and matched within ±2 months if category spanned > 4 months; Figure S3), (2) cumulative months of non-ADF ER/LA exposure (±2 months), and (3) initiator type. Initiator type was categorized as new initiator, direct switch (ADF) vs continuation (non-ADF ER/LA), or delayed (> 7-day gap) switch (ADF) vs reinitiation (non-ADF ER/LA),^46^ accounting for IR history of no IR use (ie, traditional new user) or prevalent IR use with or without a treatment gap (Figure S4). If a selected control had a history of opioid overdose or OUD before the selected prescription, that individual was excluded from further control selection, and another control was selected in place of the excluded individual.^45^ This process was repeated until there was 1 comparator non-ADF ER/LA prescription event for each individual in the ADF subcohort. Comparators were selected without replacement except for stratifications where a small sample size required selection with replacement.

In primary analyses, we used an approach similar to per protocol,^51^ where we followed patients from baseline until the first of (1) outcome of interest, (2) death (competing risk), (3) > 30 days without evidence of the treatment of interest (censoring event), (4) treatment switch (censoring event), (5) disenrollment (censoring event), (6) 180 days after the index date (censoring event), or (7) administrative censoring on September 30, 2018.

### Patient characteristics

We identified patient characteristics relative to baseline, as defined above. Baseline patient characteristics included demographics (age, sex). We identified comorbid conditions, specifically cancer, any substance use disorder, depression, obesity, and respiratory/metabolic conditions, using Elixhauser Index code groupings^52^ with a 6-month lookback. We used a derived clinical indication of acute pain, chronic pain, or surgery, identified using *ICD-9-CM* and *ICD-10-CM* codes for pain diagnoses and *CPT* codes for invasive surgery, with a 30-day lookback period to associate the derived clinical indication with the index prescription. Other prescriptions included benzodiazepines and selective serotonin reuptake inhibitors within 6 months.

In contrast to previous studies, we also identified whether the ER/LA opioid prescription was concurrently prescribed with an IR opioid,^53^ classifying episodes where the index prescription overlapped with an IR for ≥ 7 days (or the duration of the opioid prescription of interest if the ADF/non-ADF ER/LA episode was < 7 days) as concurrent. We also identified cumulative morphine milligram equivalent exposure from the first opioid prescription to the index ADF or non-ADF ER/LA prescription of interest (Appendix S1).^53^

### Statistical analysis

We used stabilized inverse probability of treatment weighting (IPTW) to account for measured confounding of the relationship between ADF use and opioid overdose or OUD. The propensity score was estimated using logistic regression to model the likelihood of treatment given measured confounders (defined in Patient characteristics, above), stratified by time since initiating non-ADF ER/LAs (in months, categorized as above) and initiator type. Confounders were identified a priori using a directed acyclic graph (Figure S5). We examined balance between treatment groups using standardized mean differences.^54^ To account for informative censoring, we used time-varying stabilized inverse probability of censoring weights (IPCW). We multiplied IPTW by IPCW to get IPTCW, truncating weights at 0.1 and 10 to minimize the influence of extreme weights.^55^

We examined the relationship between ADFs and outcomes of interest. First, we used the cumulative incidence function accounting for competing risks to estimate unweighted and weighted cumulative incidence. We used Fine-Gray^56^ models to estimate unweighted and weighted hazard ratios (HR_w_s) with the Efron approximation for tied event times. We used Schoenfeld’s residuals to test the proportional hazards assumption and considered stratifications by time if violated. We calculated 95% confidence intervals (CIs) using robust standard errors to account for clustered observations.

### Sensitivity analyses

We conducted several sensitivity analyses to evaluate the impact of analytic decisions. First, we used an approach similar to intent to treat (ITT),^51^^,^^57^ where patients were followed until the first of (1) outcome, (2) death (competing risk), (3) disenrollment (censoring event), (4) 180 days after the index date (censoring event), or (5) administrative censoring.

Next, we examined the relationship between ADFs and the combined outcome of opioid overdose or incident OUD diagnosis among the subset of individuals identified as new initiators of ADFs or non-ADF ER/LAs without prior IR opioid exposure (ie, traditional new users of opioids; Figure S4). By restricting the patient population to new initiators only, this sensitivity analysis is an example of using strict inclusion criteria to examine a single treatment strategy, comparing initiation of ADFs to initiation of non-ADF ER/LAs among individuals without a history of opioid exposure.

Finally, given that OUD is notoriously difficult to accurately measure in claims data,^58^ we conducted 2 sensitivity analyses to examine the impact of OUD misclassification. First, we excluded OUD outcomes occurring in the first 30 days of follow-up, assuming that these may not have been incident OUD outcomes. Second, we included medications for OUD (MOUD) in our definition of OUD (Table S4), excluding individuals with a history of OUD using this new definition and including additional OUD events after index based on diagnosed OUD or initiation of MOUD.

Data management was completed in SAS 9.4 (SAS Institute), and analyses were conducted in R 3.6.0 (packages listed in Table S5).^59^

## Results

### Patient characteristics

We identified 7969 patients initiating ADFs, of whom 2260 (28.4%) were classified as traditional new users, and 5709 (71.6%) were classified as patients with prior opioid exposure (Table 1). For the group of patients to serve as comparators, we identified 8888 patients initiating non-ADF ER/LAs contributing 48 797 observations used for the selection of 7969 comparator observations to match exposure histories of ADF patients (defined in Comparator selection, above).

**Table 1:** Opioid treatment history of individuals initiating ADFs or initiating, reinitiating, or continuing ER/LA opioids enrolled in private health insurance from a large provider in North Carolina, 2010-2018.

	**Non-ADF ER/LA** ***n* = 7969**	**ADF ER/LA** ***n* = 7969**
Traditional new user (> 6 months with no opioid claims)	2260 (28.36)	2260 (28.36)
Concurrent IR		
No concurrent IR	1431 (63.32)	1431 (63.32)
Concurrent IR	829 (36.68)	829 (36.68)
Prior opioid exposure (≤ 6 months since last opioid claim)	5709 (71.64)	5709 (71.64)
Switch		
Direct switch from ER/LA	426 (7.46)	426 (7.46)
Direct switch from IR	3429 (60.06)	3429 (60.06)
Delayed switch from ER/LA	161 (2.82)	161 (2.82)
Delayed switch from IR	1693 (29.65)	1693 (29.65)
Opioid treatment history		
ER/LA and IR	894 (15.66)	894 (15.66)
ER/LA only	20 (0.35)	20 (0.35)
IR only	4795 (83.99)	4795 (83.99)
Concurrent IR		
No concurrent IR	2711 (47.49)	2711 (47.49)
Concurrent IR	2998 (52.51)	2998 (52.51)
Months since first ER/LA		
0	4874 (85.37)	4874 (85.37)
1-3	216 (3.78)	216 (3.78)
4-6	114 (2)	114 (2)
7-9	79 (1.38)	79 (1.38)
10-12	70 (1.23)	70 (1.23)
13-17	100 (1.75)	100 (1.75)
18+	256 (4.48)	256 (4.48)
Months since first opioid		
1-3	1747 (30.6)	2178 (38.15)
4-6	855 (14.98)	905 (15.85)
7-9	640 (11.21)	572 (10.02)
10-12	419 (7.34)	335 (5.87)
13-17	503 (8.81)	406 (7.11)
18+	1545 (27.06)	1313 (23)
Average daily dosage (MME)		
Average MME, median (IQR)	40 (27-60)	44 (30-64)

Abbreviations: ADF, abuse-deterrent formulation of opioid analgesics; ER/LA, extended-release/long-acting formulations of opioid analgesics; IQR, interquartile range; IR, immediate-release formulation of opioid analgesics; MME, morphine milligram equivalent.

The median age at baseline was similar between the ADF (51 years; interquartile range [IQR], 41-57) and non-ADF ER/LA groups (50 years; IQR, 41-57) (Table 2). ADF initiators were less likely to be female (45.2% vs 49.6%) and were less likely to have received benzodiazepines (31.2% vs 34.8%) within 6 months before baseline. However, patients initiating an ADF were more likely to have had recent invasive surgery (42.1% vs 14.0%), an acute pain diagnosis (41.4% vs 24.5%), or a chronic pain diagnosis (80.1% vs 71.4%). Patients initiating an ADF were also less likely to have several baseline comorbidities, including depression (19.8% vs 22.1%), metastatic cancer (10.6% vs 13.3%), or solid tumor without metastasis (15.3% vs 19.1%) but were more likely to have obesity (14.4% vs 12.1%). Covariates were well balanced after implementing IPTW (Figure S6).

**Table 2:** Baseline characteristics of individuals initiating ADFs or initiating, reinitiating, or continuing ER/LA opioids enrolled in private health insurance from a large provider in North Carolina, 2010-2018.

	**Non-ADF ER/LA**	**ADF ER/LA**
*n*	7969	7969
Age, median [IQR], y	50 [41, 57]	51 [41, 57]
Female, *n* (%)	3954 (49.6)	3600 (45.2)
Index year, *n* (%)		
2010	701 (8.8)	440 (5.5)
2011	1166 (14.6)	1090 (13.7)
2012	1162 (14.6)	1083 (13.6)
2013	1053 (13.2)	1143 (14.3)
2014	1125 (14.1)	1191 (14.9)
2015	1109 (13.9)	1228 (15.4)
2016	784 (9.8)	924 (11.6)
2017	635 (8.0)	652 (8.2)
2018	234 (2.9)	218 (2.7)
Other medications, *n* (%)		
Benzodiazepines	2773 (34.8)	2486 (31.2)
SSRIs	1501 (18.8)	1462 (18.3)
Pain/surgery, *n* (%)		
Surgery	1114 (14.0)	3356 (42.1)
Acute pain	1950 (24.5)	3303 (41.4)
Chronic pain	5692 (71.4)	6382 (80.1)
Elixhauser comorbidity, *n* (%)		
Congestive heart failure	213 (2.7)	191 (2.4)
Cardiac arrhythmia	822 (10.3)	806 (10.1)
Valvular disease	308 (3.9)	252 (3.2)
Pulmonary circulation disorders	161 (2.0)	173 (2.2)
Peripheral vascular disorders	336 (4.2)	297 (3.7)
Hypertension uncomplicated	2924 (36.7)	3020 (37.9)
Hypertension complicated	260 (3.3)	296 (3.7)
Paralysis	92 (1.2)	75 (0.9)
Other neurological disorders	327 (4.1)	258 (3.2)
Chronic pulmonary disease	1139 (14.3)	975 (12.2)
Diabetes uncomplicated	1115 (14.0)	1006 (12.6)
Diabetes complicated	346 (4.3)	317 (4.0)
Hypothyroidism	837 (10.5)	759 (9.5)
Renal failure	280 (3.5)	234 (2.9)
Liver disease	676 (8.5)	623 (7.8)
Peptic ulcer disease excluding bleeding	85 (1.1)	86 (1.1)
AIDS/HIV	17 (0.2)	14 (0.2)
Lymphoma	153 (1.9)	156 (2.0)
Metastatic cancer	1060 (13.3)	841 (10.6)
Solid tumor without metastasis	1520 (19.1)	1221 (15.3)
Rheumatoid arthritis/collagen	729 (9.1)	502 (6.3)
Coagulopathy	313 (3.9)	295 (3.7)
Obesity	967 (12.1)	1149 (14.4)
Weight loss	543 (6.8)	413 (5.2)
Fluid and electrolyte disorders	1055 (13.2)	913 (11.5)
Blood loss anemia	84 (1.1)	92 (1.2)
Deficiency anemia	347 (4.4)	316 (4.0)
Alcohol use disorder	264 (3.3)	269 (3.4)
Substance use disorder	274 (3.4)	203 (2.5)
Psychoses	78 (1.0)	74 (0.9)
Depression	1763 (22.1)	1581 (19.8)
Total Elixhauser groups, median [IQR]	2 [1, 4]	2 [0, 3]

Abbreviations: ADF, abuse-deterrent formulation of opioid analgesics; ER/LA, extended-release/long-acting formulations of opioid analgesics; IQR, interquartile range; SSRI, selective serotonin reuptake inhibitor.

### Follow-up and events

In our primary analysis, patients were followed for a median of 60 days (IQR, 40-178 days; Table S6), with non-ADF ER/LA patients contributing more follow-up (median 74 days; IQR, 52-182) than ADF initiators (median 50 days; IQR, 39-115 days) due to a higher proportion of ADF patients being censored for treatment switching and/or > 30 days without ADFs (per-protocol censoring). Across all observations, there were 18 fatal or nonfatal opioid overdoses (ADF: *n* = 8, non-ADF ER/LA: *n* = 10) and 235 incident OUD diagnoses (ADF: *n* = 91, non-ADF ER/LA: *n* = 144). A competing risk of death occurred for 4% of observations in each outcome analysis.

### Model results

**Figure 1:**
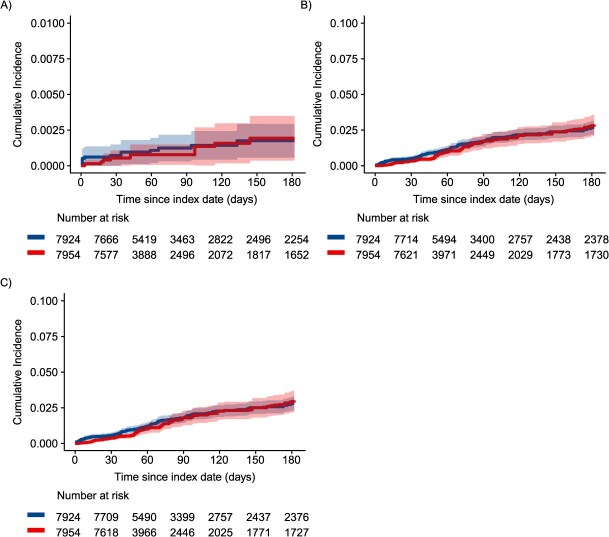
Inverse probability-weighted cumulative incidence of (A) nonfatal or fatal opioid overdose, (B) OUD, and (C) combined outcome of OUD or opioid overdose. Among individuals initiating ADF ER/LAs (red) compared to those initiating, reinitiating, or continuing non-ADF ER/LA (blue) opioids in North Carolina, 2010-2018, using an approach similar to per-protocol. ADF, abuse-deterrent formulation of opioid analgesics; ER/LA, extended-release/long-acting formulations of opioid analgesics; OUD, opioid use disorder.

Cumulative incidence of opioid overdose was consistently higher among patients who initiated, restarted, or continued non-ADF ER/LAs through the first 3 months of follow-up compared to patients who initiated ADFs in both crude (Figure S7a) and IPTCW (Figure 1A) analyses, although with considerable confidence interval overlap. Similarly, cumulative incidence of OUD was consistently higher through the first 3 months of follow-up among patients who initiated, restarted, or continued non-ADF ER/LAs compared to patients who initiated ADFs in both crude (Figure S7b) and IPTCW (Figure 1B) analyses, with the curves converging later in follow-up. Interestingly, the separation between the cumulative incidence curves is much greater in the first 6 weeks of follow-up compared to 7 weeks and later, particularly after weighting to account for confounding and informative censoring.

**Figure 2:**
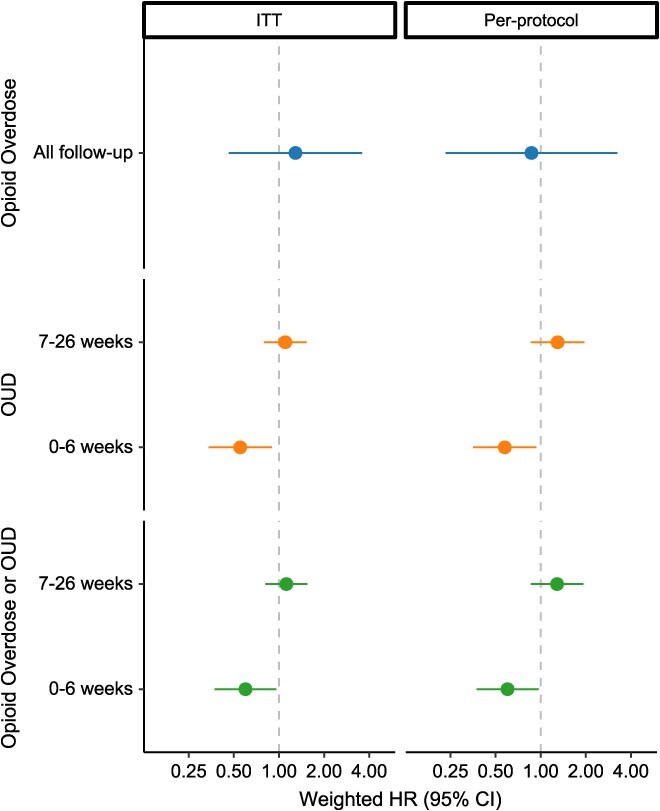
Inverse probability-weighted hazard ratios for nonfatal or fatal opioid overdose (blue), opioid use disorder (orange), and combined outcome of opioid use disorder or opioid overdose (green). Comparing individuals initiating ADF ER/LAs to those initiating, reinitiating, or continuing non-ADF ER/LA opioids in North Carolina, 2010-2018, using ITT (left) and per-protocol (right) approaches. ADF, abuse-deterrent formulation of opioid analgesics; ER/LA, extended-release/long-acting formulations of opioid analgesics; HR, hazard ratio; ITT, intent to treat; OUD, opioid use disorder.

In Fine-Gray models of opioid overdose outcomes (Figure 2, Table S7), the weighted hazard of nonfatal or fatal opioid overdose among patients initiating ADFs was 0.87 (95% CI, 0.23-3.24) times as high as the hazard among patients who initiated, restarted, or continued non-ADF ER/LAs, respectively. Tests indicated that the proportional hazards assumption for OUD outcome models was not upheld, suggesting the need to report separate estimates for the first 6 weeks of follow-up vs the remainder of follow-up. Among patients initiating ADFs, the weighted hazard of incident OUD was 0.58 (95% CI, 0.35-0.93) times as high as among patients who initiated, restarted, or continued non-ADF ER/LAs in the first 6 weeks of follow-up and 1.30 (95% CI, 0.86-1.95) times as high through the rest of follow-up (Table S8). We observed a similar relationship for the combined outcome of OUD or opioid overdose.

### Sensitivity analyses

**Figure 3:**
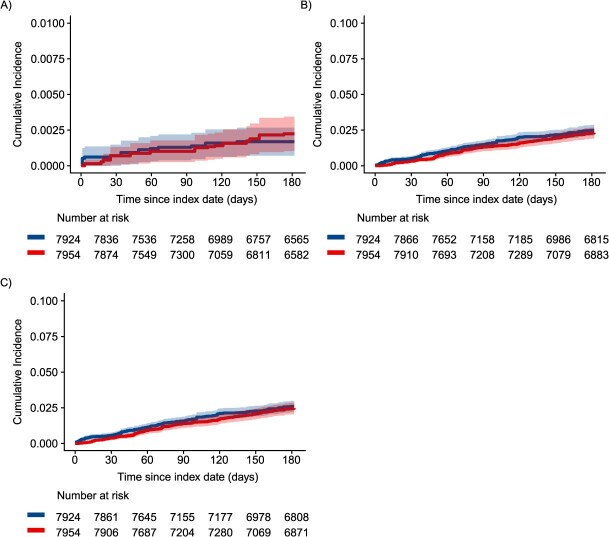
Inverse probability-weighted cumulative incidence of (A) nonfatal or fatal opioid overdose, (B) OUD, and (C) combined outcome of OUD or opioid overdose. Among individuals initiating ADF ER/LAs (red) compared to those initiating, reinitiating, or continuing non-ADF ER/LA (blue) opioids in North Carolina, 2010-2018, using an approach similar to intent to treat. ADF, abuse-deterrent formulation of opioid analgesics; ER/LA, extended-release/long-acting formulations of opioid analgesics; OUD, opioid use disorder.

In the first sensitivity analysis using an ITT approach, patients were followed for a median of 6 months, and there were 29 fatal or nonfatal opioid overdoses and 347 incident OUD diagnoses (Table S9). In this ITT analysis, we again found that the cumulative incidence of opioid overdose was consistently higher among patients who initiated, restarted, or continued non-ADF ER/LAs compared to patients who initiated ADFs through the first 3 months of follow-up (although with considerable CI overlap), with curves converging later in follow-up (Figure 3A, Figure S8). We also observed a similar relationship for OUD outcomes between the per-protocol and ITT analyses, with the greatest separation between cumulative incidence curves occurring early in follow-up (Figure 3B, C). In regression models, the weighted hazard of opioid overdose among patients initiating ADFs was 1.29 (95% CI, 0.46-3.58) times as high as among patients who initiated, restarted, or continued non-ADF ER/LAs (Figure 2, Table S7). Further, the weighted hazard of incident OUD was 0.55 (95% CI, 0.34-0.90) times as high as among patients who initiated, restarted, or continued non-ADF ER/LAs in the first 6 weeks of follow-up and 1.10 (95% CI, 0.79-1.53) times as high through the remainder of follow-up (Figure 2, Table S8).

Second, we restricted our analysis to patients who met the criteria of traditional new users (*n* = 4520) and examined the combined outcome of opioid overdose or OUD. In this subcohort, there were 39 and 45 events within 6 months of follow-up using per-protocol and ITT approaches, respectively. We found that ADFs were associated with a lower risk of opioid overdose or OUD through 6 months of follow-up using both per-protocol (HR_w_ = 0.37; 95% CI, 0.14-1.01; Figures S9-S10) and ITT approaches (HR_w_ = 0.44; 95% CI, 0.19-1.01; Figures S11-S12).

Third, there were 62 OUD events (ADF subcohort: *n* = 28) occurring in the first 30 days of follow-up and 58 OUD events (ADF subcohort: *n* = 27) occurring in the first 30 days when considering the combined outcome of OUD and opioid overdose, which were excluded from the analytic cohort for this sensitivity analysis. In this restricted subcohort, there were 173 incident OUD diagnoses (Tables S10-S11).

In the final sensitivity analysis including MOUD in the definition of OUD, there were 23 individuals who had evidence of MOUD (ADF: *n* = 6, non-ADF ER/LA: *n* = 17) before cohort entry and were thus excluded from the analytic cohort for this sensitivity analysis. In this subcohort restricted to individuals without evidence of MOUD before cohort entry, there were 237 incident OUD diagnoses or initiation of MOUD (Tables S10-S11). Results from these sensitivity analyses were consistent with results from primary analyses (Figures S13-S15).

## Discussion

In this large cohort of patients exposed to ER/LA opioids from 2010 through 2018 in NC, we examined the incidence of fatal or nonfatal opioid overdoses and diagnosed OUD, comparing patients who initiated ADFs to patients initiating, restarting, or continuing non-ADF ER/LA opioids. While the cumulative incidence of opioid overdose was lower in the ADF subcohort through the first 3 months of follow-up, there was considerable confidence interval overlap. Further, there was no notable relationship between ADF use and opioid overdose through 6 months of follow-up in regression models. We additionally found that ADF use was associated with a lower risk of OUD early in follow-up. Interestingly, while the hazard of OUD was significantly lower for patients prescribed ADFs compared to patients prescribed non-ADF ER/LAs for the first 6 weeks of follow-up, the direction of this relationship changed later in follow-up, with the hazard of OUD higher 7 weeks through 6 months among patients exposed to ADFs compared to non-ADF ER/LAs.

Several studies have sought to address whether the introduction of ADFs, which tend to be more expensive than non-ADF ER/LA opioids,^60^ was effective in reducing opioid-related harms using varied study designs,^42^ but this growing body of evidence has yielded mixed results.^17^^,^^18^^,^^20^^,^^30^^,^^61^^-^^65^ Our findings are consistent with 1 large study on OxyContin reformulation^40^ commissioned by the manufacturer using Medicaid and commercial claims data. That study found no meaningful difference in overdose rates in general but noted somewhat lower overdose among patients not taking other opioid medications concurrently. However, 78% of patients receiving ER/LA opioids are also prescribed IR opioids,^53^ limiting interpretation. The other large study on this topic,^5^ which used electronic health records, found increased overdose and OUD diagnoses in the ADF oxycodone cohort but only used IR oxycodone as the comparison group.^66^ A study of an ADF morphine product^38^ in Medicaid found higher overdose and OUD diagnoses in the non-ADF ER/LA group but had no adjustment for confounding. Given the complexity of evaluating ADFs in a postmarketing setting, there have been multiple calls to critically evaluate and improve upon the methods used to conduct postmarketing surveillance studies of ADFs.^15^^,^^41^^,^^42^^,^^67^

When designing observational postmarketing studies, key considerations include the choice of study design, appropriate comparators, accounting for confounding by “indication,” and selection bias.^41^^,^^42^^,^^58^^,^^67^ Many of these studies examined only a single ADF formulation instead of all ADFs as a class, thereby limiting generalizability. These choices influence the reliability of conclusions that can be drawn from observational studies.^66^ Our goal in this study was to implement a methodologically rigorous approach to estimate the relationship between ADFs and opioid-related harms in a population of patients we believed to be representative of the general clinical population of patients who are prescribed ADF opioids while accounting for confounding by “indication” through comparator selection and propensity score–based methods. In primary analyses, our finding of no clear relationship between ADF use and opioid overdose and a short-term reduction in risk of OUD compared to non-ADF ER/LAs was supported by multiple sensitivity analyses and is consistent with much of the growing evidence base.

Our primary analysis combined patient populations with heterogeneous treatment histories, including those who had no history of exposure to opioid analgesics, patients who had prior IR opioid exposure but no non-ADF ER/LA exposure, and patients with prior non-ADF ER/LA use. This approach allows us to examine the relationship between ADF exposure and opioid-related harms in varying treatment strategies that attempt to more closely capture real-world prescribing trajectories: (1) directly switch from the comparator (ie, non-ADF ER/LA) to the drug of interest (ie, ADF), (2) switch from the comparator to the drug of interest after a treatment gap (ie, delayed switch), or (3) initiate the drug of interest without prior exposure to the comparator (ie, new initiator).^46^ However, combining these different patient populations adds analytic complexity due to different anticipated confounding structures given heterogeneous treatment histories. We attempt to address this complexity in our weighting strategy by modeling the probability of initiating ADFs stratified by treatment history.^46^ Our sensitivity analysis restricting the population to patients without prior opioid exposure is an example of an analysis that utilizes a narrow, explicit set of inclusion criteria to examine a single treatment comparison: initiation of ADFs vs non-ADF ER/LAs among patients without prior opioid exposure. This sensitivity analysis yielded similar results to our primary analysis. Further, in our main analyses, we present average effect estimates of ADF initiation on opioid-related harm. Effect measure modification by treatment history is possible, and future studies that explicitly examine the question of modification of the relationship between ADF initiation and opioid-related harms by treatment history would be valuable contributions to the evidence base.

There are several additional limitations to consider when interpreting the findings from this study. First, opioid overdose and OUD are notoriously difficult to measure in claims data.^68^^,^^69^ Linking death records allowed us to examine fatal opioid overdoses in our study population. Additionally, we conducted 2 sensitivity analyses of OUD outcomes, incorporating MOUD into our outcome definition and excluding OUD outcomes in the first 30 days in the event that these represented prevalent OUD diagnoses rather than incident events, with results consistent with findings from primary analyses. Second, while enrollment requirements and washout periods can exclude eligible patients^70–72^ and claims data are an imperfect source of historical information about a patient’s treatment history, we chose to impose specific enrollment and washout requirements because it was important to ensure that we could capture a patient’s full treatment history with opioid analgesics (since their most recent washout period) when implementing the study design outlined in this article. We believe that treatment history is an important factor in the relationship between ADF use and opioid-related harm, and by ensuring that we were capturing a more complete treatment history, we sought to minimize the risk of measurement error of prior prescribed opioid analgesic exposure. Third, while we used IPTW to control for measured confounding, there remained the potential for unmeasured confounding. Fourth, many patients disenrolled before the end of follow-up or were censored after 30 days without evidence of an ADF or non-ADF ER/LA prescription in the per-protocol analysis. That extended-release opioids are being used for such short durations has not been previously documented. We used IPTCW to account for informative censoring and included a secondary analysis with an ITT approach to evaluate the impact of the per-protocol assumption on our findings, which produced similar results. Fifth, we caution against drawing firm conclusions about the relationship between ADF use and opioid overdose, given that these estimates had low precision. Finally, our data source included privately insured patients in NC and may not be representative of patients with Medicaid, Medicare, or those without insurance or in other regions of the country.

Even though the current problems with overdose in the United States are more closely linked to illicitly manufactured opioids (eg, street fentanyl), ADFs of novel opioid analgesics, stimulants, and cannabinoids are currently under development by pharmaceutical companies. We humbly suggest that the methods described in this study can provide a starting point for observational studies of real-world impacts of ADFs on the intended patient population.

## Conclusions

Our study aimed to answer the growing call to improve the methodological approaches in opioid safety research. In this sample of privately insured patients prescribed ER/LA opioids in NC, we used a methodologically rigorous study design to evaluate the relationship between ADFs and opioid-related harms among patients with variable opioid treatment histories and found no relationship between ADF use and opioid overdose. Interestingly, we found that ADF use was associated with a lower risk of OUD early in follow-up, but this relationship did not hold after 6 weeks, suggesting a possible limited short-term benefit from ADFs. These findings add to the expanding body of evidence that there is not a clear long-term reduction in harm from ADFs. Future work will continue to evaluate the implications of design and analytic choices in opioid safety research.

## Acknowledgments

We are grateful to colleagues at the FDA Office of Surveillance and Epidemiology for conceptual discussions that shaped our thinking on this analysis. We also thank research support staff at UNC who helped make the science possible, specifically Maryalice Nocera and LaMonda Sykes.

## Supplementary material

Supplementary material is available at the *American Journal of Epidemiology* online.

## Supplementary Material

Web_Material_kwae252

## Data Availability

The insurance claims data and death records used in this study require Data Use Agreements for access.
