# Supplementary material for: Abuse-deterrent formulations and opioid-related harms in North Carolina, 2010-2018
Source: Am J Epidemiol. 2024 Aug 9;194(3):680–90. doi: 10.1093/aje/kwae252 (PMC11879559; doi:10.1093/aje/kwae252)
Supplement: Web_Material_kwae252 [file web_material_kwae252.docx]

**Abuse-deterrent formulations and opioid-related harms in North Carolina, 2010-2018**

Bethany L. DiPrete, Nabarun Dasgupta, GYeon Oh, Daniela C. Moga, Svetla Slavova, Emily Slade, Chris Delcher, Brian W. Pence, Shabbar I. Ranapurwala

**Supplementary Data**

**Appendix S1**

**Table S1.** Abbreviations

**Table S2.** ICD-9-CM and ICD-10-CM diagnostic codes used to identify opioid overdose and opioid use disorder (OUD) in claims data

**Table S3.** ICD-10 Codes used to identify fatal opioid overdose in linked death records

**Table S4**. Medications for Opioid Use Disorder (MOUD)

**Table S5.** R packages used in analyses

**Table S6.** Follow-up and outcomes by exposure status, per-protocol analysis

**Table S7.** Fine-Gray model results: fatal or nonfatal opioid overdose

**Table S8.** Fine-Gray model results: OUD and opioid overdose or OUD

**Table S9.** Follow-up and outcomes by exposure status, ITT analysis

**Table S10.** Follow-up and outcomes by exposure status, OUD sensitivity analyses, per-protocol analysis

**Table S11.** Follow-up and outcomes by exposure status, OUD sensitivity analyses, ITT analysis

**Figure S1.** Death match algorithm

**Figure S2.** Study timelines of eligibility and exposure ascertainment for (A) traditional new users, and (B) prevalent new users.

**Figure S3.** Risk set creation and comparator selection

**Figure S4.** Schematic demonstrating classification of initiator types

**Figure S5.** Directed acylic graph (DAG)

**Figure S6.** Covariate balance before and after inverse probability weighting

**Figure S7.** Crude cumulative incidence of (A) nonfatal or fatal opioid overdose, (B) OUD, or (C) combined outcome of OUD or opioid overdose among patients initiating ADFs compared to those initiating, re-initiating, or continuing ER/LA opioids - North Carolina, 2006-2018. Using an approach similar to per-protocol.

**Figure S8.** Crude cumulative incidence of (A) nonfatal or fatal opioid overdose, (B) OUD, or (C) combined outcome of OUD or opioid overdose among patients initiating ADFs compared to those initiating, re-initiating, or continuing ER/LA opioids - North Carolina, 2006-2018. Using an ITT approach.

**Figure S9**. Inverse probability weighted cumulative incidence of the combined outcome of OUD or opioid overdose among patients initiating ADFs compared to those initiating ER/LA opioids, restricted to patients without a history of IR opioids (new users of opioid analgesics) - North Carolina, 2006-2018. Using an approach similar to per-protocol.

**Figure S10**. Crude cumulative incidence of the combined outcome of OUD or opioid overdose among patients initiating ADFs compared to those initiating ER/LA opioids, restricted to patients without a history of IR opioids (new users of opioid analgesics) - North Carolina, 2006-2018. Using an approach similar to per-protocol.

**Figure S11**. Inverse probability weighted cumulative incidence of the combined outcome of OUD or opioid overdose among patients initiating ADFs compared to those initiating ER/LA opioids, restricted to patients without a history of IR opioids (new users of opioid analgesics) - North Carolina, 2006-2018. Using an approach similar to ITT.

**Figure S12**. Crude cumulative incidence of the combined outcome of OUD or opioid overdose among patients initiating ADFs compared to those initiating ER/LA opioids, restricted to patients without a history of IR opioids (new users of opioid analgesics) - North Carolina, 2006-2018. Using an approach similar to ITT.

**Figure S13.** Inverse probability-weighted cumulative incidence of (A) OUD, excluding OUD outcomes in the first 30 days of follow-up, (B) combined outcome of OUD or opioid overdose, excluding OUD outcomes in the first 30 days of follow-up, (C) OUD, including MOUD in the definition of OUD, and (D) combined outcome of OUD or opioid overdose, including MOUD in the definition of OUD.

**Figure S14.** Inverse probability-weighted cumulative incidence of (A) OUD, excluding OUD outcomes in the first 30 days of follow-up, (B) combined outcome of OUD or opioid overdose, excluding OUD outcomes in the first 30 days of follow-up, (C) OUD, including MOUD in the definition of OUD, and (D) combined outcome of OUD or opioid overdose, including MOUD in the definition of OUD.

**Figure S15.** Inverse probability-weighted hazard ratios for sensitivity analyses: (A) OUD, excluding OUD outcomes in the first 30 days of follow-up, (B) combined outcome of OUD or opioid overdose, excluding OUD outcomes in the first 30 days of follow-up, (C) OUD, including MOUD in the definition of OUD, and (D) combined outcome of OUD or opioid overdose, including MOUD in the definition of OUD.

**References**

**Appendix S1**

*Defining opioid overdoses*

Outcomes of interest were (1) first of fatal opioid overdose, identified using ICD-10 codes for underlying and contributing cause of death in linked death records (**Table S2**), or nonfatal opioid overdose, (2) diagnosed OUD, and (3) a combined outcome of first of opioid overdose (fatal or nonfatal) or diagnosed OUD. When identifying opioid overdoses, we considered fatal opioid overdoses alone or in combination with other substances. Further, when identifying nonfatal opioid overdoses, we included events where there was at least one diagnosis code indicating opioid overdose, not excluding those who may have experienced polysubstance overdose involving an opioid in combination with other substances.

*Calculating Daily MME*

To handle overlapping prescriptions of the same ingredient when calculating MME, we assumed that in the case of a prescription with a start date ≤7 days before the end date of the previous prescription, this reflected an early refill. In this case, we pushed the start date of that prescription to the end date of the previous prescription.^1,2^ Otherwise, we treated the prescriptions as overlapping.

Next, to calculate to MME, we multiplied the dose and quantity indicated in the prescription’s National Drug Code and then divided this quantity by the prescription days’ supply recorded in the outpatient pharmaceutical claim record. We then multiplied the resulting milligrams of medication/day by the appropriate MME conversion factor obtained from CDC conversion tables.^3^ We calculated daily MME for each calendar day as the sum of MMEs per day across all prescriptions a patient had on that day.

**Table S1. Abbreviations**

| Abbreviation | Meaning |
| --- | --- |
| ADF | abuse-deterrent formulation |
| CI | confidence interval |
| ER/LA | extended-release/long-acting |
| CPT | Current Procedural Terminology |
| FDA | US Food and Drug Administration |
| HR | hazard ratio |
| ICD-9-CM | International Classification of Diseases Clinical Modification, version 9 |
| ICD-10-CM | International Classification of Diseases Clinical Modification, version 10 |
| IPCW | inverse probability of censoring weights |
| IPTW | inverse probability of treatment weights |
| IPTCW | inverse probability of treatment and censoring weights |
| IQR | interquartile range |
| IR | immediate-release |
| IRB | Institutional Review Board |
| ITT | intent-to-treat |
| MME | morphine milligram equivalent |
| MOUD | medications for opioid use disorder |
| NC | North Carolina |
| OUD | opioid use disorder |
| NDC | National Drug Code |
| SSRI | selective serotonin reuptake inhibitors |
| RD | risk difference |
| SUD | substance use disorder |
| US | United States |

**Table S2. ICD-9-CM and ICD-10-CM diagnostic codes used to identify opioid overdose and opioid use disorder (OUD) in claims data**

|  | ICD-9-CM | ICD-10-CM |
| --- | --- | --- |
| Opioid Overdose | |  |
|  | 965.00 | T40.1X1A, T40.1X4A |
|  | 965.01 | T40.0X1A, T40.0X4A |
|  | 965.02 | T40.2X1A, T40.2X4A |
|  | 965.09 | T40.3X1A, T40.3X4A |
|  | E850.0 | T40.4X1A, T40.4X4A |
|  | E850.1 | T40.601A, T40.604A |
|  | E850.2 | T40.691A, T40.694A |
|  |  |  |
| Opioid Use Disorder (OUD) | |  |
|  | 304.00 | F11.10 |
|  | 304.01 | F11.120, F11.121, F11.122, F11.129 |
|  | 304.02 | F11.14 |
|  | 304.03 | F11.150, F11.151, F11.159 |
|  | 304.70 | F11.181, F11.182, F11.188 |
|  | 304.71 | F11.19 |
|  | 304.72 | F11.20 |
|  | 304.73 | F11.220, F11.221, F11.222, F11.229 |
|  | 305.50 | F11.23 |
|  | 305.51 | F11.24 |
|  | 305.52 | F11.250, F11.251, F11.259 |
|  | 305.53 | F11.281, F11.282, F11.288 |
|  | 970.10 | F11.29 |
|  | E9350 | F11.90 |
|  | E9351 | F11.920, F11.921, F11.922, F11.929 |
|  | E9352 | F11.93 |
|  | E9401 | F11.94 |
|  |  | F11.950, F11.951, F11.959 |
|  |  | F11.981, F11.982, F11.988 |
|  |  | F11.99 |
|  |  | T40.0X1D, T40.0X2D, T40.0X3D, T40.0X4D |
|  |  | T40.1X1D, T40.1X2D, T40.1X3D, T40.1X4D |
|  |  | T40.2X1D, T40.2X2D, T40.2X3D, T40.2X4D |
|  |  | T40.3X1D, T40.3X2D, T40.3X3D, T40.3X4D |
|  |  | T40.4X1D, T40.4X2D, T40.4X3D, T40.4X4D |
|  |  | T40.601D, T40.602D, T40.603D, T40.604D |
|  |  | T40.691D, T40.692D, T40.693D, T40.694D |
|  |  | T40.0X5A, T40.0X5D |
|  |  | T40.2X5A, T40.2X5D |
|  |  | T40.3X5A, T40.3X5D |
|  |  | T40.4X5A, T40.4X5D |
|  |  | T40.605A, T40.605D, T40.695A, T40.695D |

**Table S3. ICD-10 Codes used to identify fatal opioid overdose in linked death records**

| Underlying Cause^a^ |  | Contributing Cause |
| --- | --- | --- |
| X40 |  | T40.0 |
| X41 |  | T40.1 |
| X42 |  | T40.2 |
| X43 |  | T40.3 |
| X44 |  | T40.4 |
| X60 |  | T40.6 |
| X61 |  |  |
| X62 |  |  |
| X63 |  |  |
| X64 |  |  |
| X85 |  |  |
| Y10 |  |  |
| Y11 |  |  |
| Y12 |  |  |
| Y13 |  |  |
| Y14 |  |  |

^a^The death must have a both an underlying cause code and contributing cause code.

**Table S4.** Medications for opioid use disorder (MOUD)

| Medication Name |
| --- |
| Bunavail |
| Buprenorphine HCl |
| Buprenorphine-Naloxone |
| Suboxone |
| Subutex |
| Zubsolv |

**Table S5**. R packages used in analyses

| Package | Version |
| --- | --- |
| broom | 0.5.5 |
| cobalt | 4.1.0 |
| cowplot | 1.0.0 |
| dplyr | 0.8.5 |
| forcats | 0.5.1 |
| ggplot2 | 3.3.0 |
| ggpubr | 0.2.5 |
| ggsci | 2.9 |
| ggstance | 0.3.5 |
| glue | 1.4.1 |
| haven | 2.2.0 |
| here | 0.1 |
| Hmisc | 4.4-0 |
| knitr | 1.29 |
| lattice | 0.20-38 |
| lubridate | 1.7.10 |
| magrittr | 1.5 |
| MASS | 7.3-54 |
| nnet | 7.3-12 |
| rlang | 0.4.11 |
| rms | 5.1-4 |
| stringr | 1.4.0 |
| survival | 3.2-13 |
| survminer | 0.4.6 |
| tableone | 0.11.1 |
| tibble | 2.1.3 |
| tidyr | 1.0.2 |

**Table S6.** Follow-up and outcomes by exposure status, per-protocol analysis

|  | Overall | | ADF | | ER/LA | |
| --- | --- | --- | --- | --- | --- | --- |
| Outcome | N | Median follow-up in days (IQR) | N | Median follow-up in days (IQR) | N | Median follow-up in days (IQR) |
| Analysis: Fatal or non-fatal opioid overdose | 15,938 | 60 (40-178) | 7,969 | 50 (39-115) | 7,969 | 74 (52-182) |
| Opioid overdose | 18 | 34 (17.5-87) | 8 | 33.5 (18.5-101.25) | 2,436 | 182 (182-182) |
| Administrative censoring (end of follow-up) | 4,038 | 182 (182-182) | 1,602 | 182 (182-182) | 396 | 56 (32-100) |
| Death (competing risk) | 625 | 53 (31-97) | 229 | 50 (30-89) | 549 | 93 (56-127) |
| Disenroll | 927 | 91 (55-127) | 378 | 88.5 (54-128.75) | 10 | 34 (8.5-64.25) |
| Per-protocol censoring | 10,330 | 50 (40-60) | 5,752 | 44 (36-60) | 4,578 | 60 (45-70) |
| Analysis: OUD | 15,938 | 60 (40-169) | 7,969 | 50 (38-111) | 7,969 | 71 (51-182) |
| OUD | 235 | 53 (28.5-87.5) | 91 | 52 (24-87) | 144 | 54 (31.75-88) |
| Administrative censoring (end of follow-up) | 3,933 | 182 (182-182) | 1,567 | 182 (182-182) | 2,366 | 182 (182-182) |
| Death (competing risk) | 619 | 53 (31.5-96) | 225 | 51 (30-89) | 394 | 55 (32-99) |
| Disenroll | 912 | 90 (55-127) | 373 | 86 (54-128) | 539 | 92 (56-127) |
| Per-protocol censoring | 10,239 | 50 (40-60) | 5,713 | 43 (36-60) | 4,526 | 60 (45-69) |
| Analysis: Opioid overdose or OUD | 15,938 | 60 (40-169) | 7969 | 50 (38-111) | 7,969 | 71 (51-182) |
| Opioid overdose or OUD | 247 | 53 (27-87.5) | 97 | 51 (24-88) | 150 | 54 (30.25-87) |
| Administrative censoring (end of follow-up) | 3,930 | 182 (182-182) | 1,565 | 182 (182-182) | 2,365 | 182 (182-182) |
| Death (competing risk) | 615 | 53 (32-96.5) | 224 | 50.5 (30-89) | 391 | 56 (32-99.5) |
| Disenroll | 912 | 90 (55-127) | 373 | 86 (54-128) | 539 | 92 (56-127) |
| Per-protocol censoring | 10,234 | 50 (40-60) | 5,710 | 43 (36-60) | 4,524 | 60 (44.75-69) |

**Table S7.** Fine-Gray model results: fatal or nonfatal opioid overdose

| Outcome | Weighted HR (95% CI) | Unweighted HR (95% CI) |
| --- | --- | --- |
| Per-protocol analysis |  |  |
| Fatal or nonfatal opioid overdose | 0.87 (0.23 - 3.24) | 0.97 (0.31 - 3.01) |
| ITT analysis |  |  |
| Fatal or nonfatal opioid overdose | 1.29 (0.46 - 3.58) | 1.07 (0.45 - 2.56) |

Abbreviations: HR – hazard ratio; CI – confidence interval; ITT – intent-to-treat

**Table S8.** Fine-Gray model results: OUD and opioid overdose or OUD

|  | Weighted HR (95% CI) | | | Unweighted HR (95% CI) | | | |  |
| --- | --- | --- | --- | --- | --- | --- | --- | --- |
| Outcome | 0-6 weeks | | 7 weeks - 6 months | 0-6 weeks | | 7 weeks - 6 months | |  |
| Per-protocol analysis |  | |  |  | |  | |  |
| OUD | 0.58 (0.35 - 0.93) | | 1.30 (0.86 - 1.95) | 0.76 (0.51 - 1.12) | | 0.93 (0.59 - 1.46) | |  |
| Opioid overdose or OUD | 0.60 (0.37 - 0.97) | | 1.29 (0.86 - 1.92) | 0.77 (0.53 -1.13) | | 0.95 (0.61 - 1.47) | |  |
| ITT analysis |  | |  |  | |  | |  |
| OUD | | 0.55 (0.34 - 0.90) | 1.10 (0.79 - 1.53) | | 0.62 (0.39 - 0.98) | | 0.93 (0.59 - 1.46) | |
| Opioid overdose or OUD | | 0.60 (0.37 - 0.96) | 1.12 (0.81 - 1.55) | | 0.77 (0.53 -1.13) | | 0.95 (0.61 - 1.47) | |

Abbreviations: OUD – opioid use disorder; HR – hazard ratio; CI – confidence interval; ITT – intent-to-treat

**Table S9.** Follow-up and outcomes by exposure status, ITT analysis

|  | Overall | | ADF | | ER/LA | |
| --- | --- | --- | --- | --- | --- | --- |
| Outcome | N | Median follow-up in days (IQR) | N | Median follow-up in days (IQR) | N | Median follow-up in days (IQR) |
| Analysis: Fatal or non-fatal opioid overdose | 15,938 | 182 (182-182) | 7,969 | 182 (182-182) | 7,969 | 182 (182-182) |
| Opioid overdose | 29 | 59 (25-108) | 15 | 97 (25-131) | 14 | 49 (27.25-87) |
| Administrative censoring (end of follow-up) | 13,382 | 182 (182-182) | 6,755 | 182 (182-182) | 6,627 | 182 (182-182) |
| Death (competing risk) | 876 | 72 (38-121) | 397 | 76 (39-126) | 479 | 71 (36-116.5) |
| Disenroll | 1,651 | 104 (70-142) | 802 | 106.5 (71-142) | 849 | 101 (69-141) |
| Analysis: OUD | 15,938 | 182 (182-182) | 7,969 | 182 (182-182) | 7,969 | 182 (182-182) |
| OUD | 347 | 72 (38-119) | 152 | 73 (45.5-128.25) | 195 | 69 (38-114) |
| Administrative censoring (end of follow-up) | 13,098 | 182 (182-182) | 6,636 | 182 (182-182) | 6,462 | 182 (182-182) |
| Death (competing risk) | 864 | 72 (38-119) | 389 | 75 (40-125) | 475 | 71 (36-115.5) |
| Disenroll | 1,629 | 104 (70-141) | 792 | 106 (71-142) | 837 | 101 (69-141) |
| Analysis: Opioid overdose or OUD | 15,938 | 182 (182-182) | 7,969 | 182 (182-182) | 7,969 | 182 (182-182) |
| Opioid overdose or OUD | 366 | 71.5 (38-118.75) | 163 | 73 (42.5-127.5) | 203 | 69 (37-114) |
| Administrative censoring (end of follow-up) | 13,084 | 182 (182-182) | 6,627 | 182 (182-182) | 6,457 | 182 (182-182) |
| Death (competing risk) | 859 | 72 (38-119) | 387 | 75 (39.5-124.5) | 472 | 71 (36-116) |
| Disenroll | 1,629 | 104 (70-141) | 792 | 106 (71-142) | 837 | 101 (69-141) |

**Table S10.** Follow-up and outcomes by exposure status, OUD sensitivity analysis, per-protocol analysis

|  | Overall | | ADF | | ER/LA | |
| --- | --- | --- | --- | --- | --- | --- |
| Outcome | N | Median follow-up in days (IQR) | N | Median follow-up in days (IQR) | N | Median follow-up in days (IQR) |
| Sensitivity analysis: Exclude OUD outcomes within 30d | 15,876 | 60 (40-170) | 7,941 | 50 (39-112) | 7,935 | 72 (51-182) |
| OUD | 173 | 69 (49-98) | 63 | 72 (50.5-110.5) | 110 | 65.5 (43.5-97) |
| Administrative censoring (end of follow-up) | 3,933 | 182 (182-182) | 1,567 | 182 (182-182) | 2,366 | 182 (182-182) |
| Death (competing risk) | 619 | 53 (31.5-96) | 225 | 51 (30-89) | 394 | 55 (32-99) |
| Disenroll | 912 | 90 (55-127) | 373 | 86 (54-128) | 539 | 92 (56-127) |
| Per-protocol censoring | 10,239 | 50 (40-60) | 5,713 | 43 (36-60) | 4,526 | 60 (45-69) |
| Sensitivity analysis: Exclude OUD outcomes within 30d | 15,880 | 60 (40-170) | 7,943 | 50 (39-112) | 7,937 | 72 (51-182) |
| Opioid overdose or OUD | 189 | 66 (45-97) | 71 | 71 (49-106) | 118 | 63 (43-96.25) |
| Administrative censoring | 3,930 | 182 (182-182) | 1, 565 | 182 (182-182) | 2,365 | 182 (182-182) |
| Death (competing risk) | 615 | 53 (32-96.5) | 224 | 50.5 (30-89) | 391 | 56 (32-99.5) |
| Disenroll | 912 | 90 (55-127) | 373 | 86 (54-128) | 539 | 92 (56-127) |
| Per-protocol censoring | 10,234 | 50 (40-60) | 5,710 | 43 (36-60) | 4,524 | 60 (44.75-69) |
| Analysis: Include MOUD in OUD definition | 15,915 | 60 (40-169) | 7,963 | 50 (38-111) | 7,952 | 71 (51-182) |
| OUD | 237 | 53 (29-88) | 96 | 51.5 (26.25-86.5) | 141 | 55 (31-91) |
| Administrative censoring | 3927 | 182 (182-182) | 1564 | 182 (182-182) | 2363 | 182 (182-182) |
| Death (competing risk) | 619 | 53 (31.5-96) | 225 | 51 (30-89) | 394 | 55 (32-99) |
| Disenroll | 911 | 90 (55-127) | 373 | 86 (54-128) | 538 | 92 (56-127) |
| Per-protocol censoring | 10221 | 50 (40-60) | 5705 | 43 (36-60) | 4516 | 60 (44-68.25) |
| Analysis: Include MOUD in OUD definition | 15,915 | 60 (40-169) | 7,963 | 50 (38-111) | 7,952 | 71 (51-182) |
| Opioid overdose or OUD | 249 | 53 (27-88) | 102 | 50.5 (24.25-87.5) | 147 | 55 (29.5-89) |
| Administrative censoring | 3,924 | 182 (182-182) | 1,562 | 182 (182-182) | 2,362 | 182 (182-182) |
| Death (competing risk) | 615 | 53 (32-96.5) | 224 | 50.5 (30-89) | 391 | 56 (32-99.5) |
| Disenroll | 911 | 90 (55-127) | 373 | 86 (54-128) | 538 | 92 (56-127) |
| Per-protocol censoring | 10,216 | 49 (40-60) | 5,702 | 43 (36-60) | 4,514 | 60 (44-68) |

**Table S11.** Follow-up and outcomes by exposure status, OUD definition sensitivity analyses, ITT analysis

|  | Overall | | ADF | | ER/LA | |
| --- | --- | --- | --- | --- | --- | --- |
| Outcome | N | Median follow-up in days (IQR) | N | Median follow-up in days (IQR) | N | Median follow-up in days (IQR) |
| Sensitivity analysis: Exclude OUD outcomes within 30d | 15,876 | 182 (182-182) | 7,941 | 182 (182-182) | 7,935 | 182 (182-182) |
| OUD | 285 | 86 (57-128) | 124 | 91 (59.5-136.5) | 161 | 83 (55-118) |
| Administrative censoring (end of follow-up) | 13,098 | 182 (182-182) | 6,636 | 182 (182-182) | 6,462 | 182 (182-182) |
| Death (competing risk) | 864 | 72 (38-119) | 389 | 75 (40-125) | 475 | 71 (36-115.5) |
| Disenroll | 1,629 | 104 (70-141) | 792 | 106 (71-142) | 837 | 101 (69-141) |
| Sensitivity analysis: Exclude OUD outcomes within 30d | 15,880 | 182 (182-182) | 7,943 | 182 (182-182) | 7,937 | 182 (182-182) |
| Opioid overdose or OUD | 308 | 84 (54-125.25) | 137 | 89 (57-136) | 171 | 79 (53-118) |
| Administrative censoring | 13,084 | 182 (182-182) | 6,627 | 182 (182-182) | 6,457 | 182 (182-182) |
| Death (competing risk) | 859 | 72 (38-119) | 387 | 75 (39.5-124.5) | 472 | 71 (36-116) |
| Disenroll | 1,629 | 104 (70-141) | 792 | 106 (71-142) | 837 | 101 (69-141) |
| Analysis: Include MOUD in OUD definition | 15,915 | 182 (182-182) | 7,963 | 182 (182-182) | 7,952 | 182 (182-182) |
| OUD | 352 | 72.5 (38-118) | 161 | 73 (44-128) | 191 | 69 (37-110.5) |
| Administrative censoring | 13074 | 182 (182-182) | 6623 | 182 (182-182) | 6451 | 182 (182-182) |
| Death (competing risk) | 864 | 72 (38-119) | 389 | 75 (40-125) | 475 | 71 (36-115.5) |
| Disenroll | 1625 | 103 (69-141) | 790 | 106 (71-142) | 835 | 101 (69-141) |
| Analysis: Include MOUD in OUD definition | 15,915 | 182 (182-182) | 7,963 | 182 (182-182) | 7,952 | 182 (182-182) |
| Opioid overdose or OUD | 371 | 72 (37.5-118) | 172 | 73 (42.75-127.25) | 199 | 69 (36-109.5) |
| Administrative censoring | 13060 | 182 (182-182) | 6614 | 182 (182-182) | 6446 | 182 (182-182) |
| Death (competing risk) | 859 | 72 (38-119) | 387 | 75 (39.5-124.5) | 472 | 71 (36-116) |
| Disenroll | 1,625 | 103 (69-141) | 790 | 106 (71-142) | 835 | 101 (69-141) |

**Figure S1.** Death match algorithm


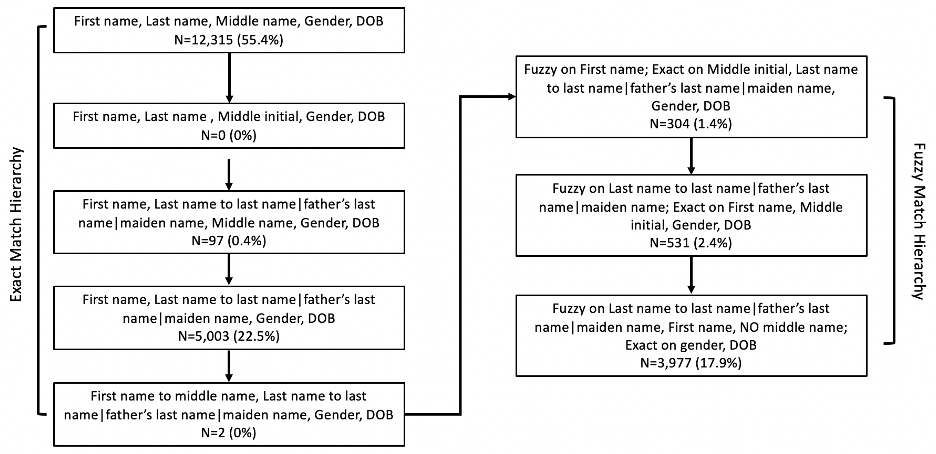


**Figure S2.** Study timelines of eligibility and exposure ascertainment for (A) traditional new users, and (B) prevalent new users.

**Figure S3.** Risk set creation and comparator selection. Months since ER/LA start categorized into 0, 1-3, 4-6, 7-9, 10-12, 13-17, 18+ months and matched within ±2 months if category spanned >4 months. One comparator (ER/LA prescription, white circles) selected per ADF patient (black circles).

**Figure S4.** Schematic demonstrating classification of initiator types

**Figure S5.** Directed acylic graph (DAG), also available at https://github.com/opioiddatalab/DAG/blob/master/DAGittyCode.md


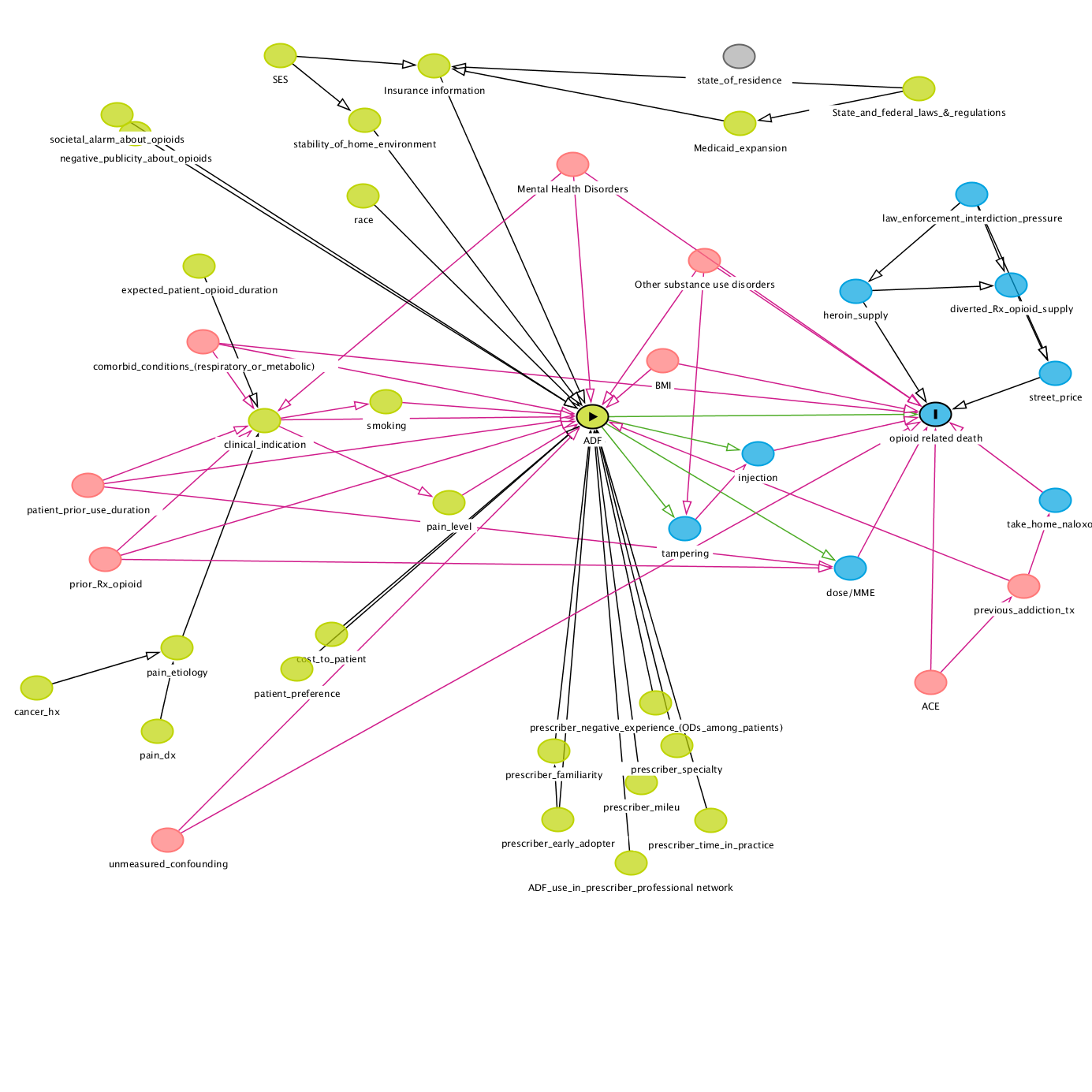


**Figure S6.** Covariate balance before and after inverse probability weighting

**
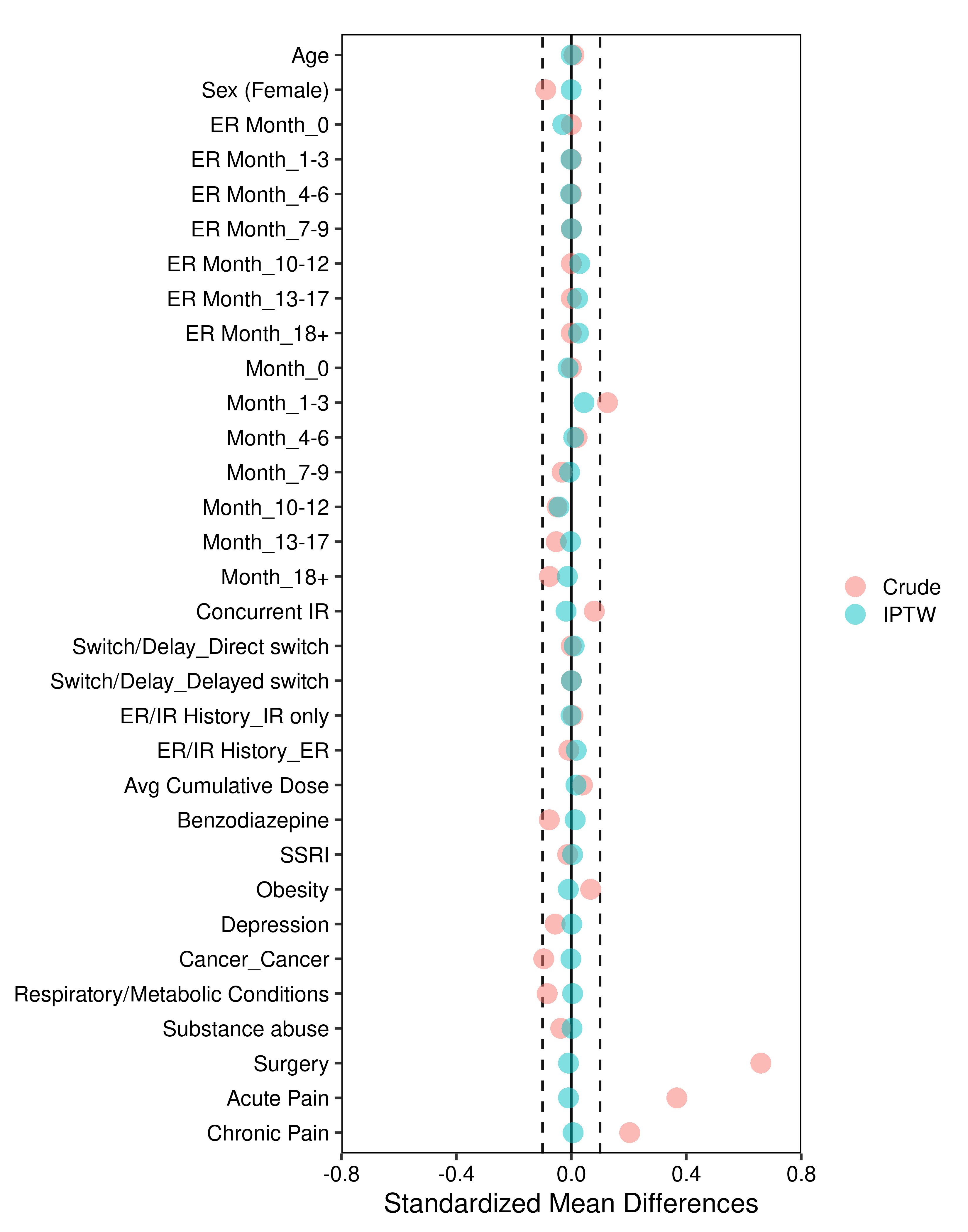
**

**Figure S7.** Crude cumulative incidence of (A) nonfatal or fatal opioid overdose, (B) OUD, or (C) combined outcome of OUD or opioid overdose among patients initiating ADFs compared to those initiating, re-initiating, or continuing ER/LA opioids - North Carolina, 2006-2018. Using an approach similar to per-protocol.

**Figure S8.** Crude cumulative incidence of (A) nonfatal or fatal opioid overdose, (B) OUD, or (C) combined outcome of OUD or opioid overdose among patients initiating ADFs compared to those initiating, re-initiating, or continuing ER/LA opioids - North Carolina, 2006-2018. Using an ITT approach.

**Figure S9.** Inverse probability weighted cumulative incidence of the combined outcome of OUD or opioid overdose among patients initiating ADFs compared to those initiating ER/LA opioids, restricted to patients without a history of IR opioids (new users of opioid analgesics) - North Carolina, 2006-2018. Using an approach similar to per-protocol.

**
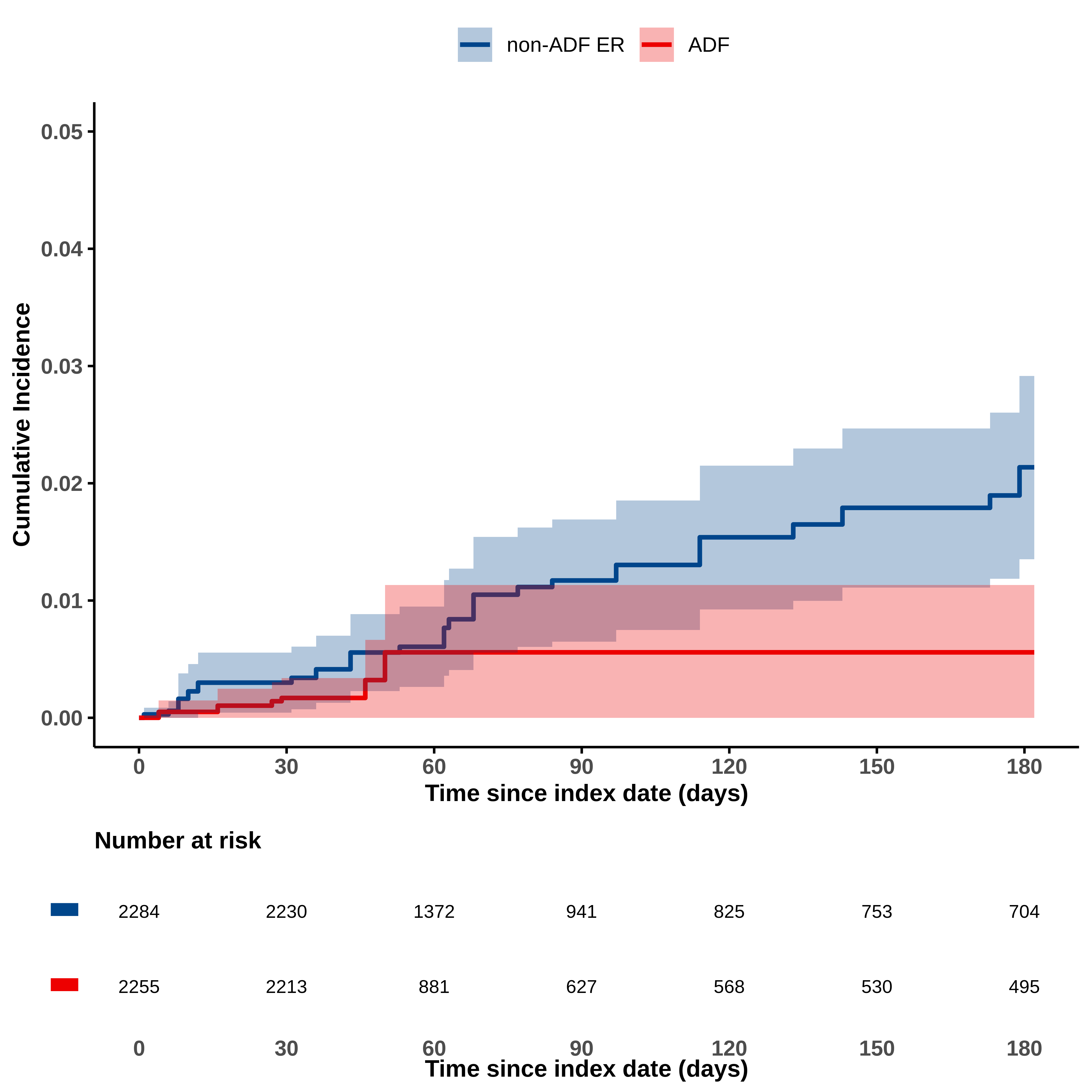
**

**Figure S10.** Crude cumulative incidence of the combined outcome of OUD or opioid overdose among patients initiating ADFs compared to those initiating ER/LA opioids, restricted to patients without a history of IR opioids (new users of opioid analgesics) - North Carolina, 2006-2018. Using an approach similar to per-protocol.

**
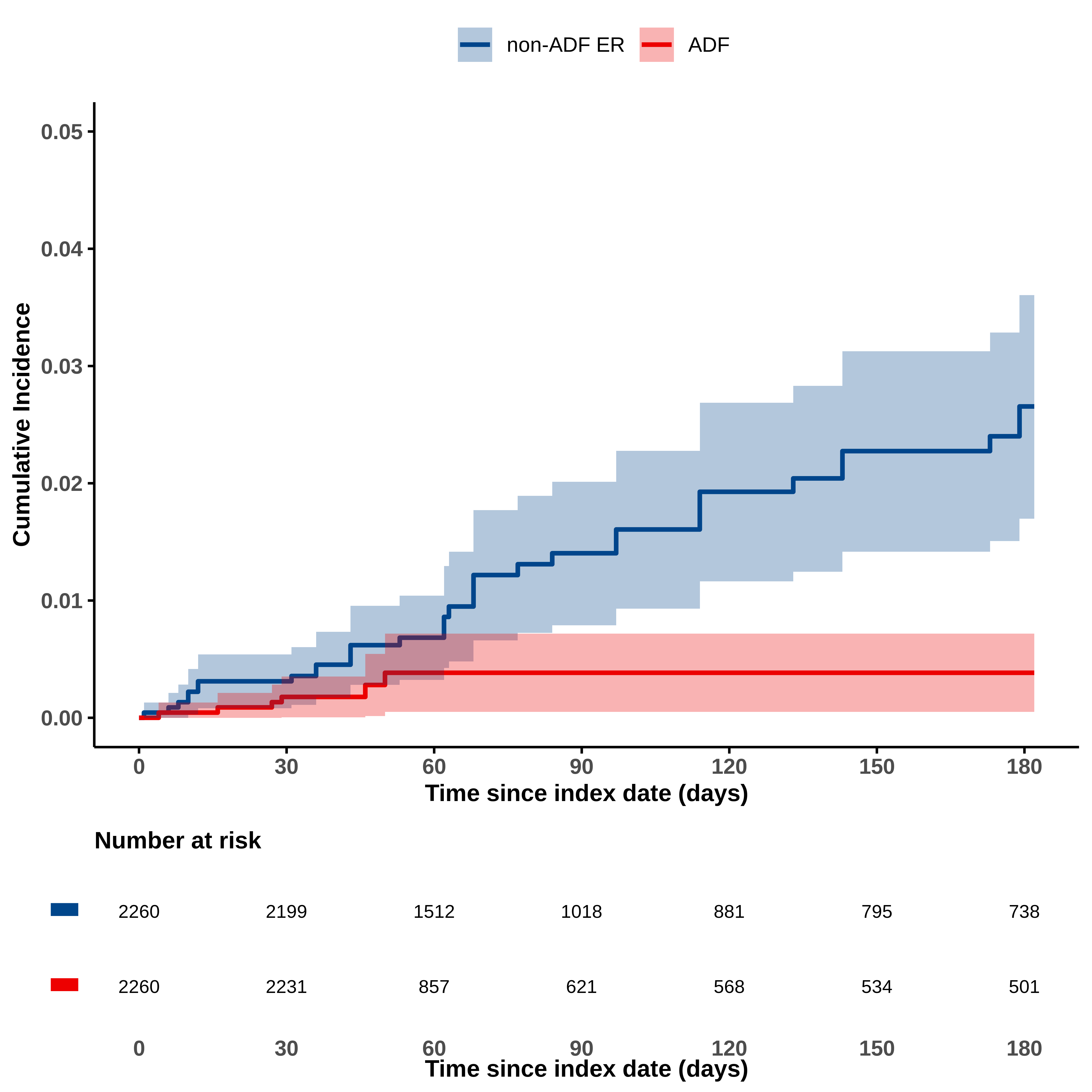
**

**Figure S11.** Inverse probability weighted cumulative incidence of the combined outcome of OUD or opioid overdose among patients initiating ADFs compared to those initiating ER/LA opioids, restricted to patients without a history of IR opioids (new users of opioid analgesics) - North Carolina, 2006-2018. Using an approach similar to ITT.

**
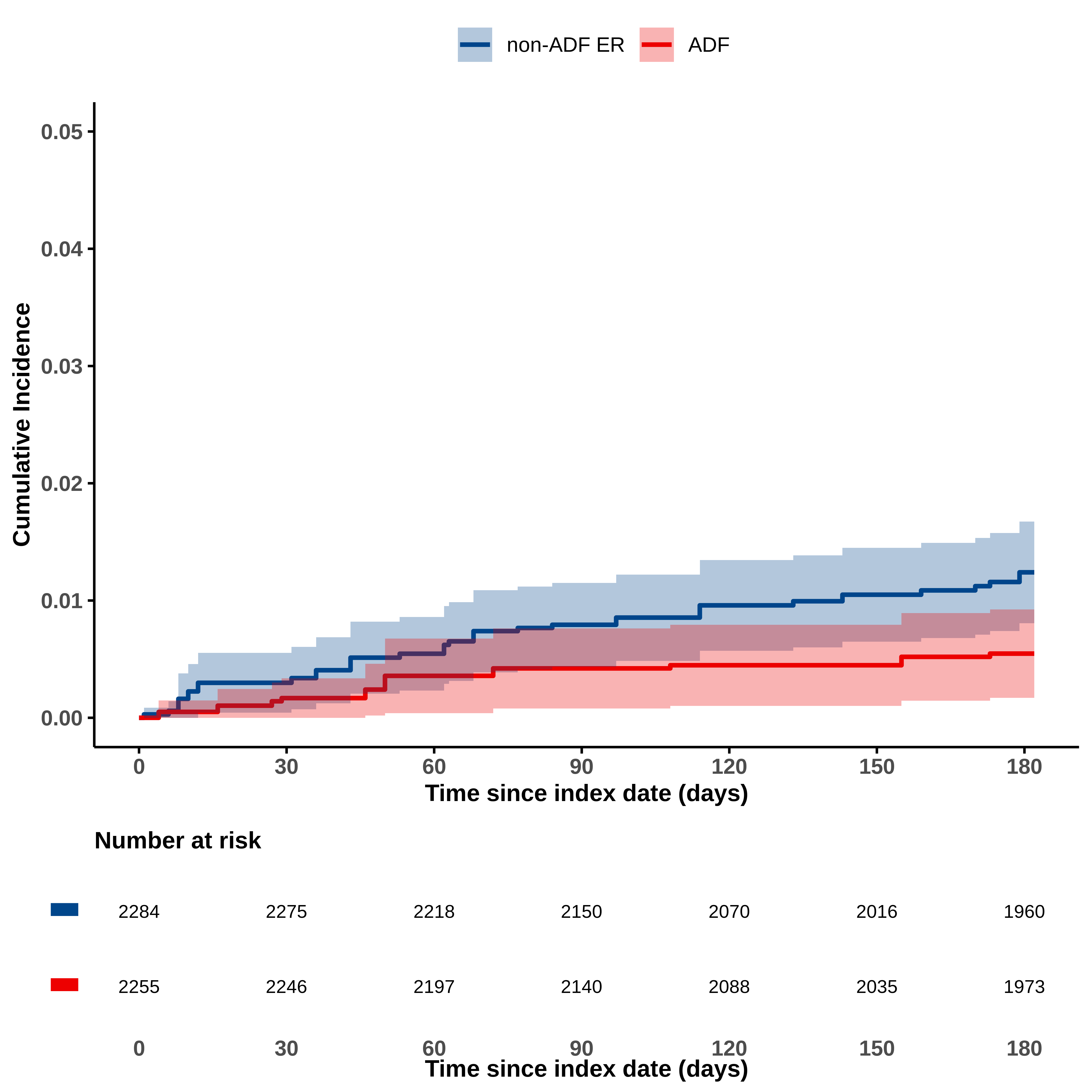
**

**Figure S12.** Crude cumulative incidence of the combined outcome of OUD or opioid overdose among patients initiating ADFs compared to those initiating ER/LA opioids, restricted to patients without a history of IR opioids (new users of opioid analgesics) - North Carolina, 2006-2018. Using an approach similar to ITT.

**
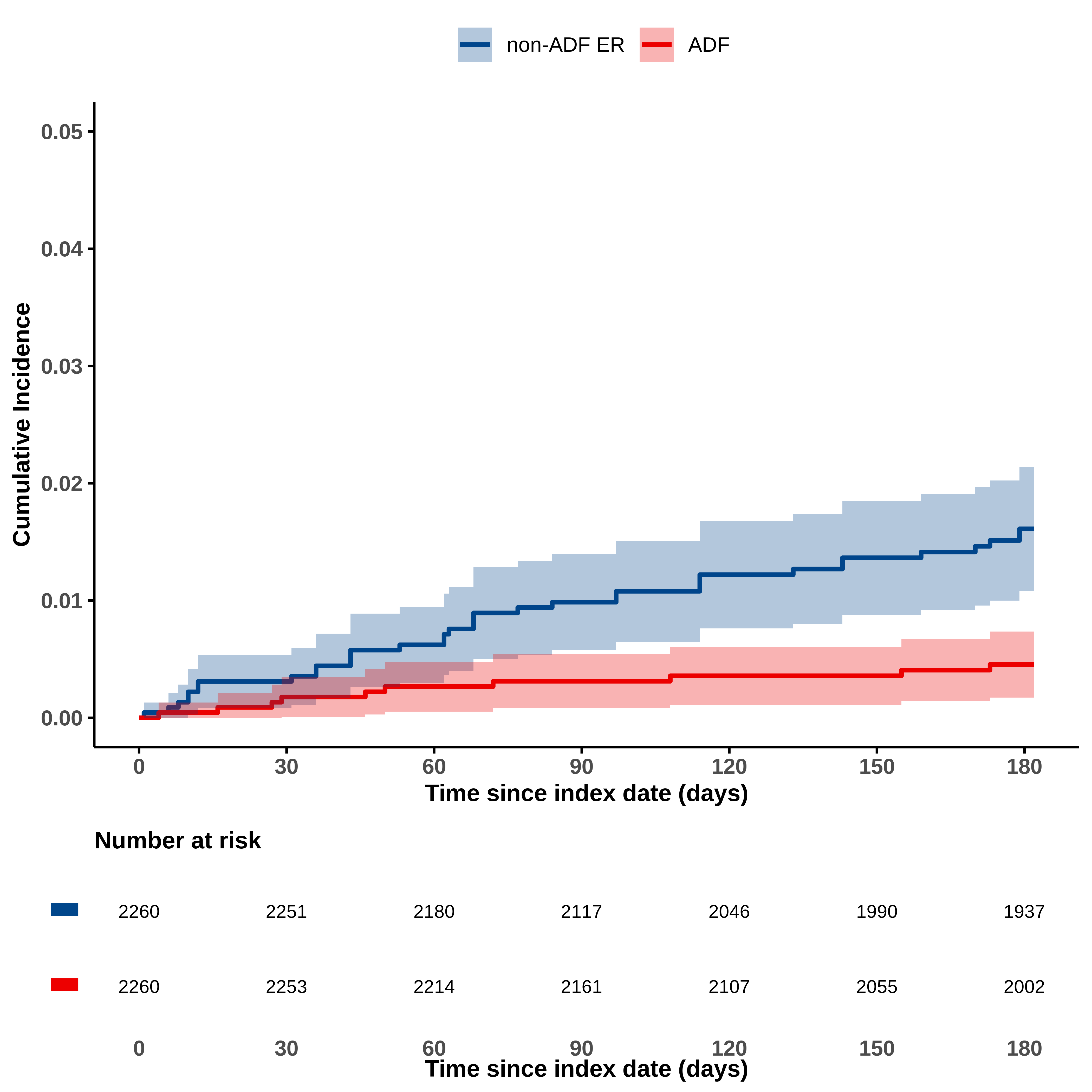
**

**Figure S13.** Inverse probability-weighted cumulative incidence of (A) OUD, excluding OUD outcomes in the first 30 days of follow-up, (B) combined outcome of OUD or opioid overdose, excluding OUD outcomes in the first 30 days of follow-up, (C) OUD, including MOUD in the definition of OUD, and (D) combined outcome of OUD or opioid overdose, including MOUD in the definition of OUD. Among patients initiating ADFs compared to those initiating, re-initiating, or continuing ER/LA opioids - North Carolina, 2006-2018. Using an approach similar to per-protocol.

**Figure S14.** Inverse probability-weighted cumulative incidence of (A) OUD, excluding OUD outcomes in the first 30 days of follow-up, (B) combined outcome of OUD or opioid overdose, excluding OUD outcomes in the first 30 days of follow-up, (C) OUD, including MOUD in the definition of OUD, and (D) combined outcome of OUD or opioid overdose, including MOUD in the definition of OUD. Among patients initiating ADFs compared to those initiating, re-initiating, or continuing ER/LA opioids - North Carolina, 2006-2018. Using an approach similar to ITT.

**Figure S15.** Inverse probability-weighted hazard ratios for sensitivity analyses: (A) OUD, excluding OUD outcomes in the first 30 days of follow-up, (B) combined outcome of OUD or opioid overdose, excluding OUD outcomes in the first 30 days of follow-up, (C) OUD, including MOUD in the definition of OUD, and (D) combined outcome of OUD or opioid overdose, including MOUD in the definition of OUD. Comparing individuals initiating ADFs to those initiating, re-initiating, or continuing ER/LA opioids in North Carolina, 2010-2018.


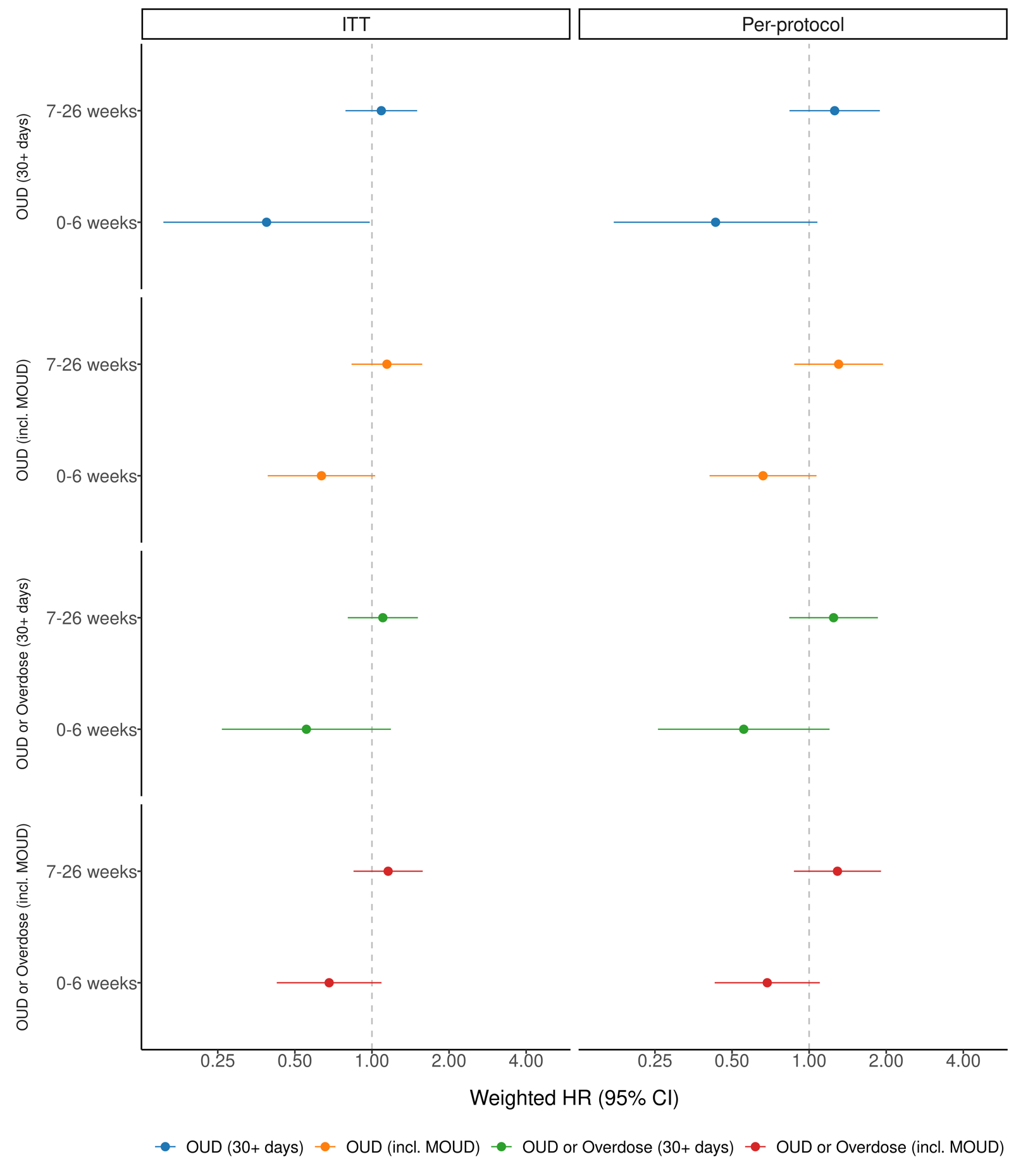


**References**

1. Ray GT, Bahorik AL, VanVeldhuisen PC, Weisner CM, Rubinstein AL, Campbell CI. Prescription Opioid Registry Protocol in an Integrated Health System. *Am J Manag Care*. May 01 2017;23(5):e146-55.

2. DiPrete BL, Ranapurwala SI, Maierhofer CN, et al. Association of Opioid Dose Reduction With Opioid Overdose and Opioid Use Disorder Among Patients Receiving High-Dose, Long-term Opioid Therapy in North Carolina. *JAMA network open*. Apr 1 2022;5(4):e229191. doi:10.1001/jamanetworkopen.2022.9191

3. Centers for Disease Control and Prevention (CDC). *National Center for Injury Prevention and Control. Opioid Overdose: Data Resources. Analyzing prescription data and morphine milligram equivalents (MME)*. 2021. Accessed July 16, 2021. <https://www.cdc.gov/opioids/data-resources/index.html>
